# A Survey of Genetic Variation and Genome Evolution within the Invasive *Fallopia* Complex

**DOI:** 10.1371/journal.pone.0161854

**Published:** 2016-08-30

**Authors:** Katarzyna Bzdega, Agnieszka Janiak, Tomasz Książczyk, Agata Lewandowska, Małgorzata Gancarek, Elwira Sliwinska, Barbara Tokarska-Guzik

**Affiliations:** 1 Department of Botany and Nature Protection, University of Silesia, Katowice, Poland; 2 Department of Genetics, University of Silesia, Katowice, Poland; 3 Department of Environmental Stress Biology, Institute of Plant Genetics, Polish Academy of Sciences, Poznań, Poland; 4 Department of Plant Genetics, Physiology and Biotechnology, UTP University of Science and Technology, Bydgoszcz, Poland; United States Department of Agriculture, UNITED STATES

## Abstract

The knotweed taxa *Fallopia japonica*, *F*. *sachalinensis* and their interspecific hybrid *F*. × *bohemica* are some of the most aggressive invaders in Europe and North America and they are serious threats to native biodiversity. At the same time, they constitute a unique model system for the creation of hybrids and studies of the initiation of evolutionary processes. In the presented study, we focused on (i) examining genetic diversity in selected populations of three *Fallopia* taxa in the invaded (Poland) and native ranges (Japan), (ii) establishing genome size and ploidy levels and (iii) identifying ribosomal DNA (rDNA)-bearing chromosomes in all of the taxa from the invaded range. We found that the genetic diversity within particular taxa was generally low regardless of their geographical origin. A higher level of clonality was observed for the Polish populations compared to the Japanese populations. Our study suggests that the co-occurrence of *F*. *sachalinensis* together with the other two taxa in the same stand may be the source of the higher genetic variation within the *F*. *× bohemica* hybrid. Some shift towards the contribution of *F*. *japonica* alleles was also observed for selected *F*. × *bohemica* individuals, which indicates the possibility of producing more advanced generations of *F*. *× bohemica* hybrids. All of the *F*. *sachalinensis* individuals were hexaploid (2*n* = 6*x* = 66; 2C = 6.01 pg), while those of *F*. *japonica* were mostly octoploid (2*n* = 8*x* = 88; 2C = 8.87 pg) and all of the *F*. × *bohemica* plants except one were hexaploid (2*n* = 6*x* = 66; 2C = 6.46 pg). Within the chromosome complement of *F*. *japonica*, *F*. *sachalinensis* and *F*. × *bohemica*, the physical mapping of the rDNA loci provided markers for 16, 13 and 10 chromosomes, respectively. In *F*. × *bohemica*, a loss of some of rDNA loci was observed, which indicates the occurrence of genome changes in the hybrid.

## Introduction

The knotweed taxa *Fallopia*: *F*. *japonica* var. *japonica*, *F*. *sachalinensis* and the hybrids of these two species, namely *F*. × *bohemica*, are considered to be one of the most aggressive plant invaders in both Europe and North America [1]. The knotweeds *F*. *japonica* and *F*. *sachalinensis*, originate from Asia. The native range of *F*. *japonica* is Japan, Sakhalin Island, the Kurile Islands, North and South Korea, Taiwan, Vietnam and parts of China, whereas for *F*. *sachalinensis* the range is limited to North Japan, Korea, South Sakhalin and the Kurile Islands [2–4]. These species were introduced into Europe in the 19^th^ century as decorative garden plants and soon spread into natural habitats [5–7]. The historical sources disclose that *F*. *japonica* was brought over from Japan to Netherlands by Phillipe von Siebold, and later it was distributed to other European countries. Some records also suggest that some specimens of this species were introduced to Great Britain from China, but probably those plants did not survived [5]. The source of *F*. *sachalinensis* was presumably the collection from St. Petersburg, where *F*. *sachalinensis* originated from Japan and Sakhalin Island where they were grown [5,6]. The first description of the occurrence of *F*. × *bohemica* hybrids in Europe was given only just in 1983 [8].

*Fallopia japonica* currently exists in most parts of the British Isles, in many parts of the European continent, also occurs in Canada and the United States, Australia and New Zealand [5,9–12]. It’s stands were confirmed in South America (Chile) [13]. The invaded range *F*. *sachalinensis* includes Europe, New Zealand as well as Australia and South Africa. This species also occurs in North America, Canada and the United States [6,12,14–16]. Both parental species, *F*. *japonica* and *F*. *sachalinensis* are particularly common in ruderal habitats that have been changed by human influence and in riparian ecosystems [6,17–19], where they create populations that are composed of one or two taxa (mostly *F*. *japonica* with *F*. × *bohemica*) or three taxa cohabiting in the same site [20,21].

Knotweeds constitute a unique model system for the creation of hybrids and studies on the initiation of evolutionary processes in an invaded range. The main hybridization routes of closely related *Fallopia* taxa are reviewed in Bailey et al. [12]. Briefly, the main taxa contributing to the hybridization process are *F*. *japonica* var. *japonica*, *F*. *japonica* var. *compacta*, *F*. *sachalinensis* and *F*. *baldschuanica*. Both varieties of *F*. *japonica* may be pollinated by *F*. *sachalinensis* producing the hybrid *F*. × *bohemica*, which subsequently may go through the rounds of backcrossing with either of parents. Both varieties of *F*. *japonica* and *F*. *sachalinensis* may also be crossed with *F*. *baldschuanica*, but there is only one established *F*. *conollyana* hybrid, resulting from the cross of *F*. *japonica* var. *japonica* and *F*. *baldschuanica* [12].

A large intraspecific ploidy variation for the *Fallopia* complex is typical in both the native and invaded ranges [22]. In the time, polyploidization and hybridization are important processes that promote genetic diversity, because recombination in hybrids generates novel variations. Ellstrand & Schierenbeck [23] postulated that higher genetic diversity in hybrid-derived populations may be responsible for their evolutionary success or invasiveness. The invasive characteristics of *F*. × *bohemica* may represent an example of an invasion via the hybridization hypothesis, which is explained as the superiority of hybrids over their parental species, which also ensures their success in the non-native range [24–26]. In a study of Parepa et al. [26], it was found that *F*. × *bohemica* produces significantly greater biomass than both of its parents, when grown in experimental communities of native plants, supporting the hypothesis that hybridization events increase the competitiveness of the *Fallopia* complex. Aside from hybridization, also multiple introductions can help to generate higher genetic diversity in the invaded range than in the native one [27,28].

The genetic diversity of a species has consequences for various processes, such as colonization, many life history traits (e.g. mode of reproduction–sexual vs. clonal, seed dispersal and life cycle), population history, the impact of environmental factors and anthropogenic disturbances [29–33].

Genetic diversity in the populations of different plant species, in and out of their native and invaded range, has been the subject of several studies [34–38]. These surveys can be useful for forecasting population’s responses to biological or chemical control measures that are based on diversity levels. Understanding the extent and distribution of the genetic diversity of invasive plants species may help to predict their response to chemical and biological eradication. Populations with limited level of genetic diversity are considered to have lower potential to evolve the resistance to herbicides or natural enemies than much more genetically diverse populations [39].

Genetic diversity studies can also indicate the origin of populations, the routes of their introduction and can explain the mechanisms of their spreading and adaptation on a local scale [40,41].

The genetic diversity in the *Fallopia* complex has been investigated using morphological, cytogenetic and molecular markers, including RAPDs, ISSRs [42–44], SNPs, SSRs [45,46] and AFLPs [47,48] as well as isoenzyme analysis [49]. However, the identification of invasive knotweeds using chromosomal markers and genome size has only been the subject of a few studies [12,50,51]. The fact that *Fallopia* taxa have small and morphological undifferentiated chromosomes makes an analysis of karyotype morphology difficult. As indicated for other genera, the application of FISH with repetitive sequences is often useful in cytogenetic analysis [52]. To the best of our knowledge, the position of ribosomal genes has not yet been documented for any *Fallopia* taxa. The preliminary chromosome identification and the dynamics of chromosome rearrangements are necessary to understand the evolution of *Fallopia* genomes, especially to identify the parental chromosomes in the *F*. × *bohemica* hybrid genome and to elucidate the extent of genomic changes that may have occurred in the *F*. × *bohemica* genome after hybridization. This genomic analysis is complementary to other molecular approaches that support comprehensive studies on a genome evolution within the invasive *Fallopia* complex.

Our hypothesis assumes that the taxa composition within a particular location may influence on the genetic diversity within the population as the presence of several taxa cohabiting together may allow their interspecific hybridization and/or backcrosses between the *F*. × *bohemica* hybrid and its parental taxa, thereby restoring fertility by enabling chromosome pairing during meiosis. We would like to testify its assumptions by focusing on the following aspects: (i) an analysis of genetic diversity in selected invasive populations of three *Fallopia* taxa from Poland and its native (Japanese) range; (ii) an analysis of the possible relationship between the level of genetic diversity within each taxon and the taxonomic constitution that is characteristic for the stands that were studied, i.e. stands consisting of one taxon (here called ‘homogeneous’) or that were composed of two or three taxa cohabiting in the same area (here called ‘heterogeneous’); (iii) an analysis of the number and chromosomal location of rDNA loci in all of the taxa from the invaded range and (iv) the genome size and ploidy of the plants collected in Poland.

## Materials and Methods

### Plant material

Three invasive knotweed taxa were used in the study: *Fallopia japonica*, *F*. *sachalinensis* and *F*. × *bohemica*. The classification of each taxon was based on their morphological characters such as leaf shape and size, trichome type and morphology, which are well described as traits that are diagnostic for the identification of *Fallopia* taxa [12]. Our study was conducted outside of protected areas, and the species plants were not subject to any kind of protection. Therefore, no specific permissions were required for any locations and activities. We also confirm that the field studies did not involve endangered or protected species.

The material was collected from three stands in Poland (PL) and from four stands in Japan (JP). In the introduced range (PL), two stands were ‘heterogeneous’–comprised of two or three taxa co-occurring in the same area and one was ‘homogeneous’ and consisted of one taxon–*F*. *japonica* (Table 1).

**Table 1:** Number of plants of each *Fallopia* population from its introduced (Poland) and native range (Japan) sampled, together with the code number used (*x*–consecutive numbers of individuals; can vary from 5 to 30).

Stands	Species	Lat. (°N)	Long. (°E)	No. of ramets per species	Code
**introduced range (Poland)**					
**Heterogeneous**					
Jasieniczanka River	FJ	49°51.020	18°55.766	30	PL_CDJ_FJ_*x*
	FB			30	PL_CDJ_FB_*x*
	FS			30	PL_CDJ_FS_*x*
Biała River	FJ	49°55.981	19°01.219	30	PL_CDB_FJ_*x*
	FB			30	PL_CDB_FB_*x*
**Homogeneous**					
Biała River	FJ	49°53.342	19°01.931	30	PL_CDB_FJ_h_*x*
**native range (Japan)**					
**Homogeneous**					
Kita-Itami, Itami, Hyogo	FJ	34°47.894	135°05.358	5	JP_It_FJ_*x*
Temma, Kita-ku, Osaka	FJ	34°41.584	135°31.230	5	JP_Os_FJ_*x*
Toma-cho, Kamikawa-gun–Hokkaido	FS	43°53.650	142°31.306	5	JP_To_FS_*x*
Obira-cho, Rumoi-gun–Hokkaido	FS	44°09.548	141°39.550	5	JP_Ob_FS_*x*

FJ–*Fallopia japonica*; FB–*F*. ×*bohemica*; FS–*F*. *sachalinensis*.

For the purpose of presented analysis, the term ‘population’ is used interchangeably with ‘taxon in a particular stand’. The stands were located along two river valleys in the southern part of Poland. All of the populations were found in natural, riparian habitats, where *Fallopia* presents the most adverse impact on local biodiversity. In the native range (JP), the samples were collected from four ‘homogeneous’ stands–two composed of *F*. *japonica* and two consisting of *F*. *sachalinensis* (Table 1). Because the ‘heterogeneous’ composition of *Fallopia* populations in Japan is not frequent, the presented analysis was concentrated on the ‘homogeneous’ populations. The populations from the native range were collected from typical stands of anthropogenic character. For the Polish populations of the study, *in situ* observations of the types of flowers and their fertility were performed. All of the individuals, from both the invaded and native ranges, were used to analyze the genetic diversity, whereas the cytogenetic analysis was limited to the individuals from the invaded range in Poland–due to legal restrictions it was not possible to get fresh plant material or seeds from the Japanese populations at the time the material was collected. To determine the genetic diversity and relationships within and between the selected populations of the *Fallopia* taxa, leaves were collected from one ramet per shoot clump (the basic unit of the rhizome system; after Bailey et al. [12]). The number of shoot clumps selected for the analysis varied from 5 to 30 for each population that was studied. A shoot clump was defined as an area of 0.5 m × 0.5 m. In order to collect ramets that represented different individual plants, a distance of more than 7 m was maintained between the shoot clumps from which material was collected [53].

Leaf tissues from 200 ramets, including five ramets from each of the populations in Japan (Itami (It), Osaka (Os), Toma-cho (To), Obira-cho (Ob)) and 30 from each Polish population from the Jasieniczanka River (CDJ) and Biała River (CDB), were used for DNA extraction and further examination using AFLP markers (Table 1).

A piece of rhizome was cut from each individual originating from the Polish populations, potted in universal potting soil, periodically and cultivated in a greenhouse at 20°C and a 14-h daily photoperiod. Newly developed root tips from this greenhouse material were used for the chromosome counts, genome size and rDNA-FISH analysis. Of the 200 individuals used for AFLP analysis, 167 were examined using flow cytometry (FCM) and 30 (10 individuals from each taxon) were used for chromosome counts and chromosome identification using 5S and 35S rDNA sequences.

### Genetic diversity analysis using Amplified Fragment Length Polymorphism (AFLP) method

#### DNA extraction and AFLP procedure

Leaf material from each ramet was collected from plants originating from native and invaded populations and was stored in silica gel (Sigma). Total genomic DNA was extracted from 100 mg of silica-dried material using the micro-CTAB method [54]. The AFLP technique followed the method of Vos et al. [55] with modifications as described by Bzdęga et al. [46]. A fluorescently labelled (IRD-800) primer for *EcoR*I cutting site was used for the further visualization of the AFLP products. To ensure reliable band pattern analysis, the entire AFLP procedure was performed in two technical replications for each sample and the fragments were separated using a denaturing polyacrylamide gel electrophoresis (6% acrylamide/bis-acrylamide 19:1 solution (Sigma), 7 M urea (Amersham Pharmacia), 1 × TBE). A Li-Cor sequencer was used for electrophoresis with the following parameters: 1300 V, 30 mA, 30 W and medium speed of laser scanning. In order to calculate the length of amplified products, a 50–350 bp size marker (50–350 bp Size Standard, Li-Cor) was loaded on to the gel. The ten most polymorphic AFLP primer combinations were used, which were selected after previously pre-screening a set of 15 selective primer pairs (S1 Table).

#### AFLP data analysis

In order to describe the level of polymorphism among the individuals, only the unambiguous bands in each primer combination were counted. The entire set of bands was transformed into a 0–1 matrix consisting of monomorphic and polymorphic products (S2 Table). The level of polymorphism was defined in two ways–as the percentage of variable loci over the total number of loci in each population and as a pairwise comparison of the percentage of polymorphic bands between each pair of individuals studied. The range of pairwise polymorphism was given and the mean pairwise polymorphism was calculated as: P = Σ (A_ij_/B_ij_ × 100) /D; where: A = the number of polymorphic loci for a pair of samples *i* and *j*, B = the total number of loci for a pair of samples analyzed and D = the number of pairs of samples analyzed. The percentage of polymorphisms within and between the populations was calculated based on the number of polymorphic loci with a minimum frequency of 5%.

The clonal diversity within populations was calculated using Simpson’s index of diversity *D* = 1 – Σ (*n*_*i*_(*n*_*i*_*−* 1)) / (*N*(*N–* 1)), where *n*_*i*_ is the number of ramets of the *i*^*th*^ genet and *N* is the total number of ramets sampled [56]. For the purpose of this analysis, a ‘genet’ was understood as a unique type of AFLP band pattern.

The expected heterozygosity (H_e_) and Nei’s index of diversity were used to measure the genetic variation among particular taxa using AFLPSURV 1.0 software [57] and an analysis of molecular variance AMOVA was performed using Arlequin 3.11 software [58].

The AFLP data was used to construct dendrogram, but only unique genets were used in order to avoid an excess of samples that would most likely represent the same clones. The dendrogram was prepared using Phylip 3.6 software [59] with a modified Nei and Li distance, the UPGMA method and 1000 replicates of bootstrapping. The resulting unrooted tree was drawn using the PhyloDraw 0.8 program [60]. Additionally, a maximum likelihood-based hybrid index (h) was calculated for each *F*. *× bohemica* individual from the population in the Jasieniczanka River, where they cohabitate with both parental taxa. The calculations were done using FAMD software [61].

Furthermore, the error rate of the AFLP analysis was estimated for each taxon. All of the bands that were produced by all of the primer combinations were calculated as the number of loci scored multiplied by the number of individuals analysed for each taxon. Then, the percentage of bands that appeared or disappeared in only one of the technical replicates was calculated.

### Genome size analysis

The genome size in the young leaves of *Fallopia* plants was estimated for 167 individuals, including 87 plants of *F*. *japonica* (from heterogeneous stands: 29 plants and 30 plants from the Jasieniczanka River and the Biała River respectively and from homogeneous stand the Biała River, 28 plants), 60 plants of *F*. × *bohemica* (from heterogeneous stands, 30 plants per each) and 20 plants of *F*. *sachalinensis* (from heterogeneous stand the Jasieniczanka River) using FCM. *Vicia villosa* cv. ‘Minikowska’ (2C = 3.32 pg/nucleus, estimated using male human leukocytes with 2C = 7 pg) was used as the internal standard. Nuclear samples were prepared as was previously described [62] using a Tris-MgCl_2_ isolation buffer (0.2 M Tris-Cl, pH 7.5, 4 mM MgCl_2_, 0.5% (v/v) Triton X-100) supplemented with 1% (w/v) PVP-10, propidium iodide (PI; 50 μg ml^-1^) and ribonuclease A (50 μg ml^-1^). For each sample, 3000–5000 nuclei were analysed immediately after preparation using a CyFlow SL Green (Partec GmbH, Münster Germany) flow cytometer, equipped with a high-grade solid-state laser with a green light emission at 532 nm, a long-pass filter RG 590 E, DM 560 A as well as with side (SSC) and forward (FSC) scatters. Histograms (CV = 3.23–5.41) were evaluated using FloMAX software (Partec GmbH, Münster, Germany). Nuclear DNA content was calculated using the linear relationship between the ratio of the *Fallopia/Vicia* peak positions on the histogram of fluorescence intensities. Additionally, the 1Cx (DNA content of one non-replicated monoploid genome with chromosome number *x*) was calculated.

### Ploidy level and rDNA loci distribution assessment

#### Chromosome preparation and counting

The root tips of ten individuals of *F*. *japonica*, *F*. × *bohemica* and *F*. *sachalinensis* selected from Polish populations were collected in ice water. They were then pretreated with 2 mM 8-hydroxyquinoline for 4 h, fixed in ethanol with glacial acetic acid (3:1, v/v) and then stored at -20°C until use. Fixed roots were washed in a 0.01 M citric acid-sodium citrate buffer (pH 4.8) and digested in an enzyme mixture of 20% (v/v) pectinase (Sigma), 1% (w/v) cellulase (Calbiochem) and 1% (w/v) cellulase ‘Onozuka R-10’ (Serva) for 2–2.5 h at 37°C. A single root tip was washed in cold distilled water and transferred into a drop of 45% acetic acid on microscope slide and squashed. The coverslips were removed after freezing and slides were air-dried. Chromosome analysis was carried out on 3–5 well-spread metaphases. Each chromosomal preparation was derived from a different single root tip, thus each preparation corresponded to one individual.

#### DNA probes

Two kinds of probes were used–(i) 5S rDNA probe was generated by the PCR amplification of a 410-bp *Bam*HI sub-clone of the 5S rDNA from the wheat clone pTa794 [63] and labelled by PCR with tetramethyl-rhodamine-5-dUTP (Roche) using the universal M13 ‘forward’ (5`-CAG GGT TTT CCC AGT CAC GA-3`) and ‘reverse’ (5`-CGG ATA ACA ATT TCA CAC AGG A-3`) sequencing primers. The thermal cycling programme was as follows: 94°C for 1 min, 35 cycles of 94°C for 40 s, 55°C for 40 s and 72°C for 90 s and finally 72°C for 5 min and (ii) 26S rDNA probe, which was used to detect the 35S rDNA loci, was made by the nick translation of a 2.3-kb *Cla*I sub-clone of the 26S rDNA coding region of *Arabidopsis thaliana* [64] with digoxigenin-11-dUTP (Roche). The conditions for this reaction were as follows: 15°C for 95 min and 65°C for 10 min.

#### Fluorescence in situ hybridization (FISH), image capturing and processing

The pre-treatment and denaturation of chromosome slides subjected to the FISH experiments were carried out as follows: RNase treatment (37°C for 1 h), 0.1× SSC at room temperature for 1 min, incubation of slides in 2× SSC at 65°C for 20 min, 0.1× SSC at room temperature for 1 min, denaturation of slides in 0.07 N NaOH at room temperature for 1 min, 0.1× SSC at 4°C for 1 min, 2× SSC at 4°C for 1 min and then the slides were dehydrated in an ethanol series at room temperature ([65], with minor modifications). The FISH procedure was performed as described in detail by Książczyk et al. [66].

All FISH images were acquired using an Olympus XM10 CCD camera attached to an Olympus BX 61 automatic epifluorescence microscope. Image processing and superimpositions were done using Olympus Cell-F imaging software and Micrographx Picture Publisher 8.0 software.

## Results

### Types of flowers and their fertility

The types and fertility of flowers were recorded for each *Fallopia* population from the invaded range. We found male sterile flowers of *F*. *japonica* in all three of the populations in Poland. Individuals of *F*. × *bohemica* from all of the populations had male fertile flowers (Biała River–heterogeneous stand) and flowers of both sexes (Jasieniczanka River–heterogeneous stand). We also recorded the presence of both sexes of *F*. *sachalinensis*–females and hermaphrodites were observed at the Jasieniczanka River. No flowers were observed in the *Fallopia japonica* and *F*. *sachalinensis* populations from Japan at the time the material for the presented study was collected.

### Genetic diversity using AFLP markers

The analysis of genetic diversity of the *Fallopia* populations from the introduced and native ranges based on AFLP markers and ten selective primer combinations resulted in a total of 679 loci of good quality and 323 (47.6%) of these were polymorphic across all of the samples in the study. These primer combinations generated 150 552 bands and the majority of those exhibited a consistent pattern in both technical replications of the AFLP analysis. Only 21 bands produced ambiguous results, resulting in an error rate of 0.0139%.

### Genetic diversity within the populations

The number of polymorphic loci that was detected for the majority of the populations studied was very low, ad did not exceed 5% (Table 2), although two exceptions were observed–the *F*. × *bohemica* population from the Jasieniczanka River in Poland was characterized by the occurrence of 17% polymorphic AFLP loci and the population of *F*. *japonica* from Itami in Japan had about 10% polymorphic loci. These values were also accompanied by the highest values of the expected heterozygosity from among all of the populations studied (Table 2).

**Table 2:** Number and frequency of polymorphic loci and expected heterozygosity (He) within *Fallopia* populations from the introduced and native ranges.

Population	No. of loci	No. loci (freq.> 5%)	Proportion of polymorphic loci (%)^a^	He	S.E. (He)
**introduced range**					
**Poland**					
PL_CDJ_FJ	675	669	4.57	0.00446	0.00087
PL_CDJ_FB	671	671	17.08	0.05233	0.00507
PL_CDJ_FS	625	625	0.29	0.00097	0.00069
PL_CDB_FJ	646	646	0.29	0.00037	0.00029
PL_CDB_FB	654	653	5.89	0.00392	0.00060
PL_CDB_FJ_h	658	648	0	0	0
**native range**					
**Japan**					
JP_It_FJ	637	637	10.31	0.05146	0.00594
JP_Os_FJ	622	622	0.74	0.00368	0.00167
JP_To_FS	568	568	3.68	0.01781	0.00357
JP_Ob_FS	565	565	3.24	0.01386	0.00296

Codes of populations–see Table 1. FJ–*Fallopia japonica*; FB–*F*. ×*bohemica*; FS–*F*. *sachalinensis*; S.E.–standard error.

^a^ Percentage of polymorphic loci with a minimum of 5% frequency.

Pairwise comparisons of polymorphism within populations were also generally low, regardless of their geographical origin (Table 3). No polymorphism was observed among the individuals of the *F*. *japonica* population from the Biała River in Poland, which represented a homogeneous stand where the only taxon that occurred was *F*. *japonica*. In the case of the remaining Polish *F*. *japonica* populations that cohabitates in the same area with other *Fallopia* taxa, the mean pairwise polymorphism ranged from 0.04% to 0.45% and the maximum polymorphism between pairs of *F*. *japonica* individuals was 3.26%. The analysis of the populations from the same taxon located in the native range showed a wider range of polymorphism from 0.41% in Osaka to 5.8% in Itami and the highest pairwise polymorphism was calculated at 6.8%.

**Table 3:** Mean pairwise polymorphism and clonal diversity (Simpson’s diversity index) within *Fallopia* populations from the introduced and native ranges.

Population	No. of individuals (ramets)	No. of genetic individuals (genets)	DNA polymorphism (%)^a^		Simpson’s diversity index
			Range	Mean value	
**introduced range**					
**Poland**					
PL_CDJ_FJ	30	5	0–3.26	0.45	0.36
PL_CDJ_FB	30	23	0–8.91	5.40	0.96
PL_CDJ_FS	30	2	0–0.32	0.11	0.37
PL_CDB_FJ	30	3	0–0.31	0.04	0.19
PL_CDB_FB	30	2	0–6.08	0.41	0.07
PL_CDB_FJ_h	30	1	0	0	0
**native range**					
**Japan**					
JP_It_FJ	5	5	1.80–6.83	5.83	1
JP_Os_FJ	5	5	0.16–0.80	0.42	1
JP_To_FS	5	5	0.54–3.53	2.23	1
JP_Ob_FS	5	4	0–3.89	1.74	0.9

Codes of populations–see Table 1. FJ–*Fallopia japonica*; FB–*F*. ×*bohemica*; FS–*F*. *sachalinensis*.

^a^ The levels of polymorphism were calculated after pairwise analysis of individuals from each taxon.

A higher polymorphism range (from 0.4 to 5.4%) was observed in *F*. × *bohemica* from Poland (Table 3). Moreover, the highest pairwise polymorphism of 8.91% was observed within this taxon. In the case of the remaining taxon, *F*. *sachalinensis*, a polymorphism of more than ten-fold higher was observed within the populations from the native range than for the introduced range.

The relatively low level of polymorphism within the three taxa resulted from their clonal reproduction, which was especially characteristic for the populations from Poland (Table 3). Each of these populations was represented by 30 individuals, which allowed the identification of individuals with the same AFLP fingerprint, referred here as belonging to the same genet. The highest number of genets was observed in the *F*. × *bohemica* population from the Jasieniczanka River, and the lowest, only one genet, within the population of *F*. *japonica* from the Biała River (homogeneous stand;Table 3). The remaining populations from native ranges were represented by much lower numbers of individuals, which hinders their precise comparison with Polish populations. Nevertheless, the values of the Simpson index of diversity, which may be translated as the probability that any randomly selected individual from the population will represent a different genet, shows that the overall clonality of the populations from outside of Poland is much lower.

### Genetic diversity between the populations

In the introduced range the lowest value of polymorphism, 2.76%, was observed between *F*. *japonica* population from the Biała River (homogeneous stand) and the population of the same taxon located along the same river valley, but which was cohabiting with individuals of *F*. × *bohemica*. Comparable level of variation, of around 5%, was found between different populations of *F*. *japonica*, from both the Biała and the Jasieniczanka Rivers as well as between *F*. *japonica* from the Biała River (homogeneous stand) and *F*. × *bohemica* population from the same river valley. The highest diversity (around 10–11%) was noticed between *F*. *sachalinensis* from the Jasieniczanka River and all other taxa from invaded range.

Similar comparisons for the populations from native range showed that the less variation was observed between two *F*. *sachalinensis* populations from Obira-cho and Toma-cho (3.75%). Higher polymorphism was present between *F*. *japonica* from Itami and Osaka (8.74%).

The highest value of polymorphic loci, 25.22%, was observed between the *F*. *japonica* population from Itami and the *F*. *sachalinensis* population from Obira-Cho in Japan.

When a comparison of populations from the introduced and native ranges was performed, it was shown that Polish and Japanese *F*. *japonica* populations differ between each other on average in 10–12% of AFLP loci. The hybrid *F*. × *bohemica* from invaded range was more polymorphic in comparison to *F*. *sachalinensis* (19.81%) than to *F*. *japonica* from native range (11–13%). The highest polymorphism was noticed between *F*. *sachalinensis* populations from Poland and Japan (19–20%; S3 Table).

The Nei’s genetic distance values also show that populations of *F*. *sachalinensis* from the native range and all of the *Fallopia* populations from Poland, including the Polish population of the same taxon, were the most genetically distinct (Nei’s genetic distance higher than 0.1727; Table 4). At the same time, two of the populations of *F*. *sachalinensis* from Japan (from Toma-cho and Obira-cho) were the most similar (Nei’s genetic distance of 0.0158). A comparison of the *Fallopia* populations from Poland showed that the genetic distance between the different *F*. *japonica* populations, between the *F*. *japonica* and *F*. × *bohemica* populations or between different populations of *F*. × *bohemica* was similar and usually lower than the distance between the population of *F*. *sachalinensis* and any of the populations of remaining taxa (Table 4).

**Table 4:** Pairwise Nei’s genetic distance between *Fallopia* populations.

Population		Introduced range						Native range		
		PL_CDJ_FJ	PL_CDJ_FB	PL_CDJ_FS	PL_CDB_FJ	PL_CDB_FB	PL_CDB_FJ_h	JP_It_FJ	JP_Os_FJ	JP_To_FS
**Introduced range**	PL_CDJ_FJ									
	PL_CDJ_FB	0.0526								
	PL_CDJ_FS	0.1045	0.0897							
	PL_CDB_FJ	0.0473	0.0712	0.1138						
	PL_CDB_FB	0.0459	0.0610	0.1068	0.0269					
	PL_CDB_FJ_h	0.0623	0.0635	0.1129	0.0526	0.0462				
**Native range**	JP_It_FJ	0.1037	0.1089	0.1660	0.1079	0.1019	0.1106			
	JP_Os_FJ	0.1025	0.1122	0.1631	0.1105	0.1007	0.1154	0.0577		
	JP_To_FS	0.2077	0.1727	0.2018	0.2114	0.2132	0.2059	0.2428	0.2349	
	JP_Ob_FS	0.2153	0.1800	0.2100	0.2226	0.2237	0.2137	0.2488	0.2409	0.0158

**Fig 1:**
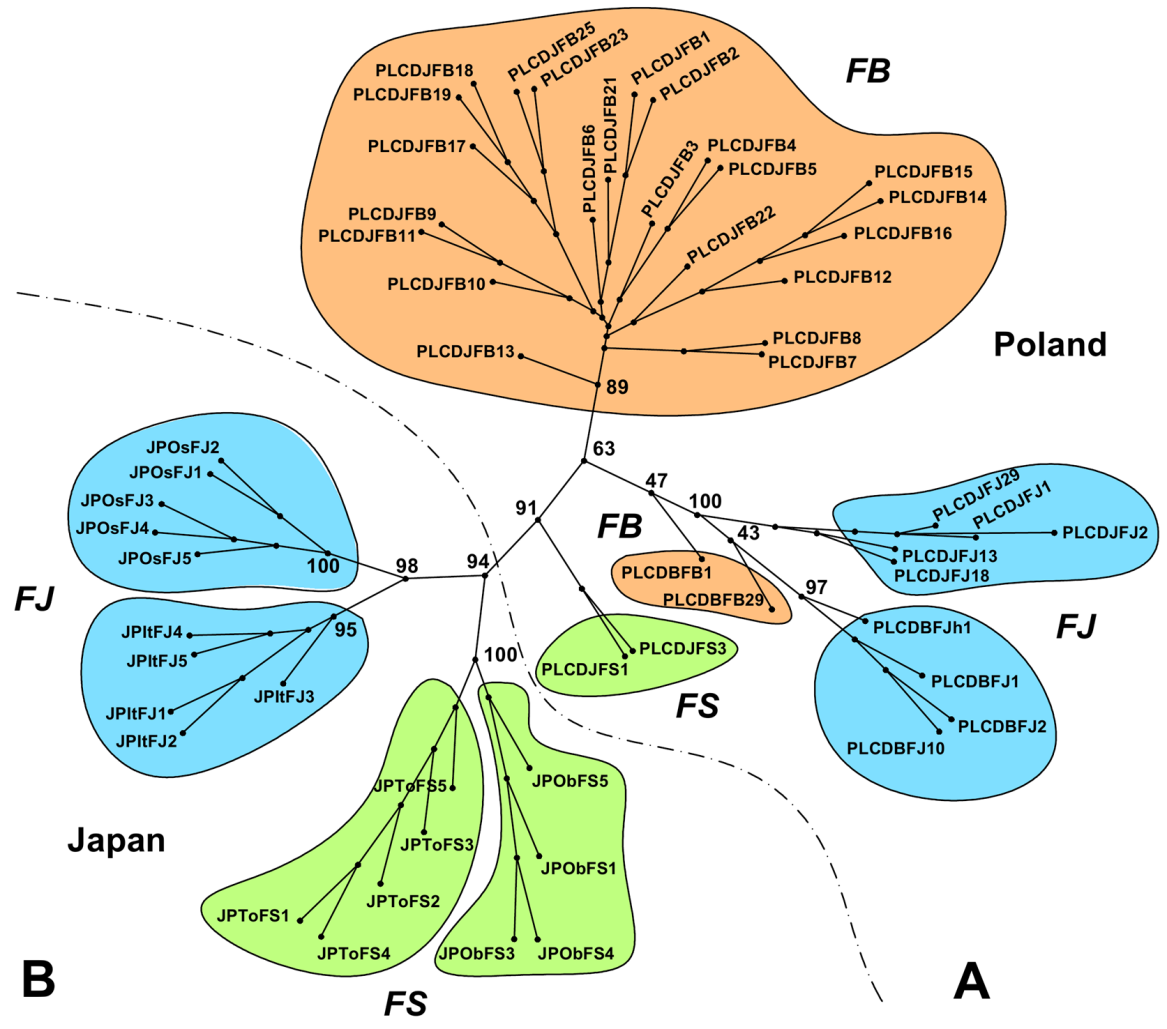
**Unrooted tree of *Fallopia* genets representing populations from its introduced (A) and native (B) ranges.** Codes of populations–see Table 1, FJ–*Fallopia japonica* (marked with blue color), FB–*F*. ×*bohemica* (marked with orange color), FS–*F*. *sachalinensis* (marked with green color); for more explanation see ‘Materials and methods‘ section. The numbers at the tips of the branches refer to individuals. The numbers next to the branch nodes refer to the bootstrap value (%). A dashed line with dots separates the populations from Poland and the remaining stands.

A dendrogram based on the AFLP data showed that all taxa were grouped into separate nodes with the exception of two genets of the *F*. × *bohemica* population from the Biała River (heterogeneous stand; PL_CDB_FB) (Fig 1). Considering the populations from Poland, located in the heterogeneous stand with all three taxa cohabiting in the same area, the individuals from the *F*. × *bohemica* population were grouped between the individuals of the parental taxa. A cluster of *F*. *japonica* genets from the Biała River was grouped close to the genets of the same taxon from the Jasieniczanka River. In addition, genets from the *F*. × *bohemica* population from the Biała River were located close to these two above-mentioned clusters. One genet showed higher similarity to the *F*. *japonica* from the Biała River and the other to the *F*. *japonica* from the Jasieniczanka River. However, the bootstrap values for their positions in the dendrogram were lower than 50%. All of the populations from Poland were well separated from the populations from Japan. The latter also formed separate clusters that included genets from particular populations from the native range.

Because a stand from the Jasieniczanka River was composed of three taxa cohabiting together, the calculation of the maximum likelihood-based hybrid index was performed for the individuals of *F*. × *bohemica* from that location using FAMD software. The results showed that the hybrid index for the majority of these individuals was near 0.5, thus indicating the equal genetic contribution of the parental alleles in the genomes of the hybrid (S4 Table). Some shift towards the contribution of *F*. *japonica* alleles was noticed for six *F*. × *bohemica* individuals (hybrid index between 0.613 and 0.658).

Partitioning of the molecular variance using hierarchical AMOVA in which all of the populations were divided into two groups based on their range (introduced or native) showed that the majority of the variance (about 65%) was explained at the among-population level. An important part of the observed variance (about 22%) was contributed by groups. The smallest amount of the observed variance resulted from the diversity within each population (Table 5). When populations were grouped according to the composition of taxa, i.e. as belonging to populations from homo- or heterogeneous stands, a negative variance component was obtained, thus indicating that no genetic structure is associated with this grouping of criteria.

**Table 5:** Partitioning of genetic variation using AMOVA analysis.

Source of variation	Degree of freedom	Sum of squares	Variance components	Percentage of variation
**Groups–country of origin (introduced and native range)**				
Among groups	1	290.698	5.01747	22.80
Among populations within groups	8	2384.117	14.31964	65.06
Within populations	190	507.700	2.67211	12.14
Total	199	3182.515	22.00921	
**Groups–‘homo’ or ‘heterogeneous’ populations**				
Among groups	1	244.815	-1.58867	-9.27
Among populations within groups	8	2430.000	16.05749	93.68
Within populations	190	507.700	2.67211	15.59
Total	199	3182.515	17.14092	

P < 0.0001

### Genome size and chromosome number

FCM analysis and chromosome counts confirmed the occurrence of hexaploid and octoploid plants among the *Fallopia* species (Table 6). It revealed that the population of *F*. *sachalinensis* contained hexaploid plants exclusively with a mean 2C = 6.01 pg, while hexaploids and octoploids occurred among the plants of *F*. *japonica* and *F*. × *bohemica*. However, the *F*. *japonica* plants were predominantly octoploid (2C = 8.87 pg) with only 3% hexaploid (2C = 6.45 pg). Ninety-eight per cent of the *F*. × *bohemica* plants were hexaploid (2C = 6.46 pg) and only one plant out of 59 was octoploid and had 2C = 8.96 pg DNA. The 1Cx DNA content of *F*. *sachalinensis* was 1.00 pg and it differed slightly for the other two species depending on the ploidy; it was 1.08 pg for hexaploids and 1.11–1.12 pg for octoploids.

**Table 6:** Ploidy and genome size of *Fallopia* species originating from Poland. For population code see Table 1.

Population	Ploidy	No. of individuals estimated by FCM	DNA content (pg)	
			2C (± SD)	1Cx
PL_CDJ_FJ	6*x*	2	6.454 ± 0.031	1.076
	8*x*	27	8.861 ± 0.096	1.108
PL_CDJ_FB	6*x*	29	6.461 ± 0.062	1.077
	8*x*	1	8.963	1.120
PL_CDJ_FS	6*x*	20	6.013 ± 0.125	1.002
PL_CDB_FJ	8*x*	30	8.886 ± 0.118	1.111
PL_CDB_FB	6*x*	30	6.457 ± 0.084	1.076
PL_CDB_FJ_h	8*x*	28	8.846 ± 0.119	1.106

FCM–Flow cytometry

### rRNA gene distribution between taxa

**Fig 2:**
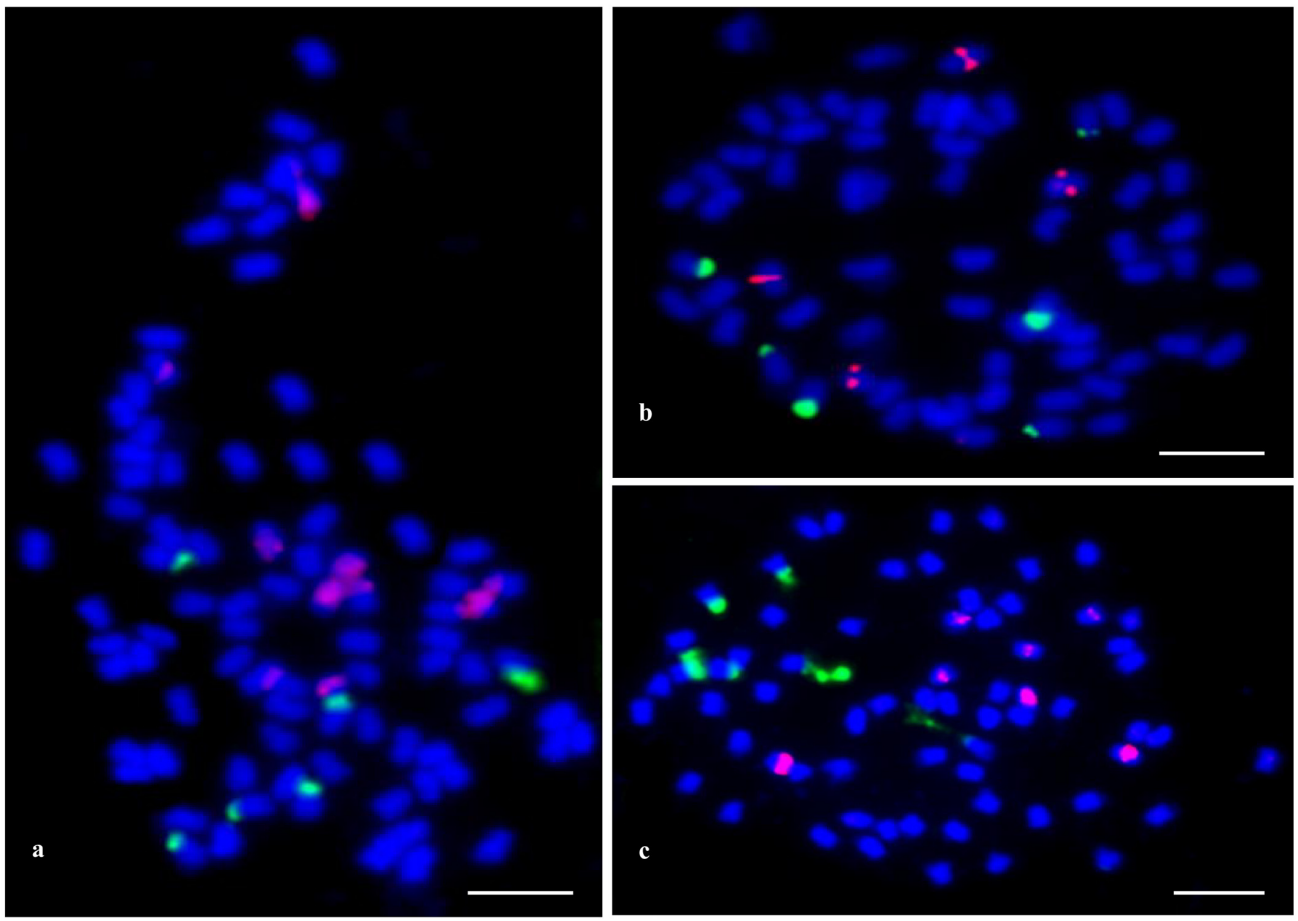
**FISH for somatic metaphase chromosomes of *F*. *japonica* (a), *F*. × *bohemica* (b), *F*. *sachalinensis* (c) showing 35S rDNA and 5S rDNA-bearing chromosomes.** FISH images were created using probes as follows: (*i*) 5S rDNA labelled with rhodamine (red) and (*ii*) 26S rDNA labelled with digoxigenin and detected by anti-digoxigenin conjugated with FITC (green); chromosomes were counterstained with DAPI (blue). Scale bars represent 5 μm.

FISH with 26S and 5S rDNA probes allowed the number and position of 35S and 5S rRNA gene loci to be determined in three *Fallopia* species (Fig 2). Double FISH with rDNA probes to the metaphase chromosomes revealed that these two types of rDNA sequences were located in different chromosomes. Among the three *Fallopia* taxa, the number and position of 35S rDNA loci were constant. All of them showed three pairs of 35S rDNA loci located in the terminal part of the chromosome arms (Fig 2A–2C). In the octoploid *F*. *japonica* individuals, all of the pairs of 35S rDNA loci were of a similar size, while in both the hexaploid *F*. *sachalinensis* and *F*. × *bohemica* plants, the 35S rDNA loci differed in size–two pairs were major and one pair was minor. Variability also occurred in the number of 5S rDNA loci and it ranged from 4 to 10 signals among the three *Fallopia* taxa (Fig 2A–2C). 5S rDNA hybridization signals were observed in five pairs of *F*. *japonica* chromosomes (two pairs of major signals were located proximally and three other minor ones located terminally; Fig 2A). In two pairs of *F*. × *bohemica* chromosomes, all pairs of 5S rDNA signals (which were of a similar size), were located in the proximal part of the chromosomes (Fig 2B). Seven signals of 5S rDNA loci were only observed in the *F*. *sachalinensis* plants, the genes of 5S rRNA were always located in the proximal part of the chromosomes and differed in size, and three signals were major and four other ones were minor (Fig 2C).

## Discussion

### Genetic variation of the knotweed complex

The analysis of genetic diversity among *Fallopia* populations from its invaded range in Poland and from its native range in Japan shows that the majority of the populations are characterized by a relatively low polymorphism. This is especially evident for the *F*. *japonica* and *F*. *sachalinensis* populations in the invaded range, in which the number of polymorphic loci was less than 1% in three of the four populations. The very low variation of *F*. *sachalinensis* may be a more general characteristic of this taxon in Poland, at least for the populations that colonize natural habitats as no polymorphism was detected in the *F*. *sachalinensis* in our previous study of the population in the Wapienica River [47]. A higher, but not very high, diversity was detected for the *F*. *sachalinensis* populations from Japan and both of the Japanese populations from our study were relatively similar to each other.

A low level of polymorphism in the Polish *F*. *japonica* populations that were studied in presented paper is in agreement with our previous analysis [47] and with the data from others. For example, Richards et al. [67] reported a low level of sequence-based genetic variation of *F*. *japonica* in and around New York, whereas several studies of *F*. *japonica* populations in Europe showed their genetic uniformity [5,12,42,44]. Regardless of the low polymorphism within *F*. *japonica* populations of presented study, our results confirm that the populations of this taxon in Poland are not represented by a single clone. Such a conclusion is supported by our cluster analysis and also by the detection of a slightly higher polymorphism in the *F*. *japonica* taxon from the Jasieniczanka River–the stand where three *Fallopia* taxa cohabitate in the same area. Similar founding of several *F*. *japonica* genets was noticed in the study of *Fallopia* populations in the United States, where they were examined using SSR markers [46].

The low level of AFLP polymorphism within the majority of the Polish *Fallopia* populations was accompanied by a high level of clonality, which is a common characteristic of *Fallopia* taxa in an invaded range and this seems to be the most predominant strategy for their reproduction [24,46]. Clonal growth is also one of the traits that describe many invasive species. An interesting model was proposed by Fukuri & Araki [68], which assumes that a spatial niche reflecting habitat heterogeneity drives a species towards a predominance of clonal propagation when a high frequency of environmental changes are presumed. The Polish populations from our study inhabit river valleys, which may be one of the reasons for the higher degree of their clonal reproduction. A riparian habitat may be subjected to many frequent disturbances that result from hydrological conditions. Human influence on the shapes of river banks or periodic flooding can cause the fragmentation of rhizomes that are further transported along the river, thus allowing colonisation of new areas via clonal spread.

A very different observation was made for the *Fallopia* populations from Japan in the presented study. Although they were represented by a lower number of individuals, the Simpson’s index of diversity shows that the probability that any randomly selected individual from these populations will represent a different genet is at least 90%. This illustrates the importance and predominance of the sexual reproduction of those taxa in their native range. Moreover, those populations were located at sites that had an anthropogenic character where different environmental factors may be important for the dynamics of those populations.

In the invaded range in Poland, one exception from the predominance of clonal growth was found and it concerned the population of *F*. × *bohemica* from the Jasieniczanka River (PL_CDJ_FB) where this taxon cohabitates with both of its parental taxa. Because this population contained multiple genotypes and the level of clonality was very low, it raises the possibility that sexual reproduction may take place at this stand. The values of the hybrid index calculated for the individuals from this population suggest that most of them were formed *de novo* after the hybridization events of the parental taxa. The observations of the types of flowers produced by those taxa also allow for such a possibility and suggest that some individuals of *F*. *sachalinensis* were the donors of pollen for the fertilization of *F*. *japonica* flowers, which is the general way in which the *F*. × *bohemica* hybrid is formed in the invaded range [69]. Our results also imply that in some cases the production of more advanced generations of the *F*. × *bohemica* hybrid may be possible, which may result from the pollination of *F*. *japonica* flowers by *F*. × *bohemica* or by sexual reproduction among *F*. × *bohemica* individuals. These events may possibly be accompanied by the elimination of some part of the *F*. *sachalinensis* chromosomes, thus resulting in progeny with an allelic composition that is skewed towards the *F*. *japonica* parent (S4 Table). Such a possibility is also supported by our cytogenetic analysis, which is discussed later.

One of our hypotheses is that the taxa composition within a particular location may influence on the genetic diversity within the population. As indicated by the AMOVA analysis, it seems that the ‘heterogeneity’ or ‘homogeneity’ of the stand itself is not the main factor that is responsible for the degree of the observed polymorphism, especially within the *F*. × *bohemica* populations. In two of ‘heterogeneous’ locations from Poland, different levels of genetic variation were found for *F*. × *bohemica*. It was high for the population from the Jasieniczanka River and almost negligible for the population from the Biała River. The difference between these two sites is the presence of the *F*. *sachalinensis* taxon in the Jasieniczanka River, which supports the possibility that this taxon is involved in the *de novo* formation of the *F*. *× bohemica* hybrid and that these events may be the source for the increased number of multiple genotypes in the population of *F*. × *bohemica*. A question remains, what is the reproduction strategy of *F*. × *bohemica*, when there is no *F*. *sachalinensis* in the same location. One possibility, which was mentioned earlier, is that the F_1_ hybrids are able to back-cross with the *F*. *japonica* parent or to produce further generations of hybrids in which part of the *F*. *sachalinensis* genome is lost. It is possible that during one or a few generations of this type of reproduction, the subsequent generations lose the ability to reproduce sexually and switch to vegetative propagation. Such a possibility may explain the situation found in the *F*. × *bohemica* population from the Biała River that cohabitates with *F*. *japonica* where only two genets were found. The location of these genets on the dendrogram near the *F*. *japonica* populations also suggests that they are closely related to that parental taxa. Moreover, Nei’s genetic distance between the *F*. × *bohemica* population from the Biała River and all of the other *F*. *japonica* from Poland was much lower than that between this population and the population of the same taxon from the Jasieniczanka River. Such a result again supports the possibility that individuals of *F*. × *bohemica*, although retaining their morphological characteristics, are becoming genetically more similar to *F*. *japonica* over subsequent generations.

### Genome size and chromosome number

Nuclear DNA content is considered to be a reliable molecular marker for the identification of *Fallopia* taxa [50,51]. The 2C DNA content of the *Fallopia* species that had previously been established varied between 0.68 pg and 9.64 pg. The present estimates, which range from 6.013 pg to 8.963 pg, fall within this range. The 2C values reported here are about 7–9% lower than those presented by Suda et al. [51], most probably because of the different internal standard that was used, but the differences may be also due to the different locations of populations studied. A much lower (by 27%) genome size for the octoploid *F*. *japonica* was estimated by Bailey et al. [50], but as was already explained by Suda et al. [51], the reason for this was most probably the method that was used (Feulgen densitometry).

The chromosome number of the *Fallopia* species studied previously was 20, 22, 40, 44, 66 and 88 [22,50]. In this study, only hexaploids (within all taxa) and octoploids (within *F*. *japonica* and *F*. × *bohemica*) were found. The presence of tetraploids, however, was observed within the *F*. *sachalinensis* taxon in our preliminary study of a population from the Jasieniczanka River. That preliminary study was limited to seven individuals and was not combined with AFLP analysis. However, it resulted in the identification of one tetraploid and six hexaploid plants (data not shown). This finding is in contrast to the *F*. *sachalinensis* ploidy in Czech populations, which are predominantly tetraploid with some representatives of hexa- and octoploid forms [22,51]. In the remaining taxa studied here, the vast majority of *F*. *japonica* individuals were octoploid and the *F*. ×*bohemica* hybrids were predominantly hexaploid. It is probable that the hexaploid *F*. × *bohemica* originated after the hybridization of the 8x *F*. *japonica* and 4x *F*. *sachalinensis* parents, although the occurrence of 4x *F*. *sachalinensis* individuals appeared to be very rare in the population in this study. This observation also raises the question on the possible advantage of a hexaploid constitution of *F*. *sachalinensis* in the habitat that was studied.

In populations of *F*. *japonica* analyzed here, only two hexaploid plants (encoded PL_CDJ_FJ_13 and PL_CDJ_FJ_18) occurred in the octoploid population. Since no hexaploid forms of this species were found previously and the 1Cx of those plants was the same as that of *F*. × *bohemica* (1.08 pg; in *F*. *japonica* it was 1.11 pg), it can be assumed that they were hybrids rather than *F*. *japonica*. Similarly, the single octoploid plant (encoded PL_CDJ_FB_13) that was found in the hexaploid population of *F*. × *bohemica*, which had 1Cx = 1.12 pg, seems to belong to another taxa, possibly *F*. *japonica*. This finding points to the genetic identity of these individuals as measured by AFLP analysis. The comparison of their fingerprints with other samples shows that they represent genets that differ from all of the other individuals from the taxa under study. In a dendrogram, the PL_CDJ_FJ_13 and PL_CDJ_FJ_18 individuals are grouped together within the *F*. *japonica* cluster from the Jasieniczanka River, although they are placed farther from other individuals of this population. Two individuals from *F*. × *bohemica* are located at a short genetic distance from this cluster, which may indicate their relatively close genetic kinship. It may also be evidence of advanced backcrossing events between an initial hybrid and its parental species, in this case *F*. *japonica*. This could result in a common cytological constitution of Polish *F*. × *bohemica* plants being retained with some admixture of *F*. *japonica* loci, which was also reflected at the phenotypic level. A similar explanation may be the case for the PL_CDJ_FB_13 individual from the Jasieniczanka River population. Its morphology resembles *F*. × *bohemica*, but its genome size is closer to *F*. *japonica* and the dendrogram shows that it is slightly more distant from the other *F*. × *bohemica* individuals from the Jasieniczanka River population and its genet is also different than all of the other individuals from the taxa studied.

Other reports point to the existence of a heterogeneous constitution of the hybrids [7] and show that it is possible to distinguish several intermediate genetic groups among *Fallopia* taxa [69]. Such groups include ‘pure’ *F*. *japonica* and *F*. × *bohemica* individuals and also a group of *F*. × *bohemica* that is close to the *F*. *japonica* individuals. The latter group may represent successive generations of *Fallopia* amphiploids or introgression [69], which may also support the hypothesis derived from our data. Further and more detailed cytogenetic studies within the *Fallopia* complex, including a comparison of resynthesized (newly formed) and relatively recent *Fallopia* allopolyploids, might be necessary to address the question of whether genome changes following allopolyploidy are random or whether there is a predisposition to change of individual loci.

### Genome structure

The fact that *Fallopia* taxa have small and morphological undifferentiated chromosomes makes an analysis of karyotype morphology difficult. As indicated for other genera, the application of FISH with repetitive sequences is often useful in cytogenetic analysis [52]. To the best of our knowledge, the position of ribosomal genes has not yet been documented for any *Fallopia* taxa. The chromosome complement of the three *Fallopia* taxa delivered four to ten chromosomes with a locus 5S rDNA and six chromosomes with a locus 35S rDNA. None of chromosomes identified bear the fraction of rDNA at which both rDNA loci were colocalized. Double-FISH with 5S and 35S rDNA provided markers for 10–16 out of 66–88 chromosomes of the *Fallopia* taxa. A comparative study of the number and position of chromosomal rRNA genes showed some similarity between all of the genomes that were studied; this early stage of *Fallopia* comparative cytogenetics might indicate a complex phylogenetic relationship (origin) of the *Fallopia* taxa. It is worth mentioning that only three pairs of 35S rDNA-bearing chromosomes were observed in the *F*. *japonica* individuals (2*n* = 8*x* = 88). We would expect eight 35S rDNA loci to be present since each subgenome (1*x*) harbours a single rDNA locus. One possible interpretation is that a homozygous deletion of two 35S rDNA loci occurred during or after the autoalloploidization of the *F*. *japonica* octoploid stabilization.

In the case of the *F*. × *bohemica* plants, the rDNA loci polymorphism might be a proof of its spread via clonal reproduction, [50], but this probably does not explain the same number of 35S rDNA loci in all taxa that were analyzed. Some assertions for clonal reproduction in plants have already been presented [70,71], what shows that hybrids would be more inclined to adjust the rDNA loci pattern during their subsequent establishment and adaption to the new habitats. The number of 35S rDNA loci as well as their locations was very similar in both parental species. A hybridization signal of 35S rDNA was observed in the terminal part of three chromosome pairs and was detected with different intensities, thus suggesting a different copy number of basic repeats. The chromosomal organization of the 35S rDNA sequences of the parental species was also similar in the *F*. × *bohemica* genome-like chromosomes. This genome exhibited the same number of 35S rDNA loci that were observed in both parental species, although some variation in the number and position of 5S rDNA loci were revealed. Locus loss has previously been detected after allopolyploid and autopolyploid formation [72], although rDNA loci additivity (i.e. that tetraploids have twice the number of loci than diploids, for instance) is also a common finding, particularly in polyploids of a recent origin [73]. Variation in the chromosome patterns of rDNA loci is common and has also been observed in other plant species [66,74,75], thereby providing valuable information about the phylogenetic relationships between related taxa. Rearrangements of repetitive sequences during the evolution of polyploid genomes was described earlier for many species and is thought to be a part of the diploidisation process [76], i.e. a result of a genome imbalance following hybridization, the duplication of rRNA gene loci as well as the reduction of dispensable parental-genome-like sequences, which was indicated in the *F*. × *bohemica* hybrid plants. The rapid loss or *de novo* generation of NORs affects the homogenization of rDNA in natural hybrids [77]. The unexpected number of 35S rDNA loci in the *F*. × *bohemica* hybrid plants might also be mediated by the uniparental elimination of (r)DNA, which may be part of a broader process of DNA deletion that is induced by interspecific hybridization [78], especially when compared to hybrids with unequal chromosomal contributions of the parental species due to a different ploidy level. Furthermore, rDNAs may be a target of rearrangements as was shown in newly synthesized allotetraploids of *Brassica* species [79,80], thus presenting changes in the chromosomal location. The rRNA gene copy number is known to evolve quickly and it is possible that some sites are lost (at least two 35S rDNA loci are missing in the *F*. *japonica* octoploid individuals); the deletion of a few pairs of 5S rDNA loci probably also occurred in *F*. × *bohemica*. In the *F*. *sachalinensis* and *F*. × *bohemica* genomes, the main 5S rDNA loci are embedded next to centromeric heterochromatin, which is typically rich in transposable elements, and therefore a transposon-mediated rearrangement might also contribute to the loss or transposition of 5S rDNA sequences [81,82] that were observed in the octoploid *F*. *japonica*. This suggests chromosome rearrangements resulting from a genome imbalance during (auto/allo)polyploid formation as was recently shown in *Tragopogon* allotetraploids [82]. Unfortunately, there is no clear evidence as to what mechanism may be involved in 5S rDNA loci variation in the *Fallopia* plants, so further studies are required for critical FISH analyzes on the variation patterns of rDNA loci.

## Conclusions

The combination of several molecular methods used in the presented study made it possible to give a broader picture of the genetics and evolution of the invasive *Fallopia* complex. Based on a genetic diversity study, we found that significant differences exist between the populations from both the invaded and native ranges. The Polish populations of the study are characterized by a different reproduction strategy with a very high level of clonality compared with the native populations. Our cytological studies show the occurrence of genome rearrangements in *F*. *×bohemica* as indicated by the rDNA-FISH analysis where the gain or loss of rDNA loci together with some rDNA movements might be a possible trend of chromosome evolution in this genus. We also found a relatively high uniformity of genome ploidy within the taxa studied with only a few exceptions that may represent successive generations of hybrids or introgression forms of the parental species *F*. *japonica* or *F*. *sachalinensis* in the background of *F*. *×bohemica*. The findings of the presented study open another questions on genome composition and its evolution within the *Fallopia* complex. They relate to local phenomenon such as the occurrence of *F*. *sachalinensis* in the heterogeneous stands and its influence on the overall genetic diversity of taxa that cohabitate together. An interesting question also relates to the details of the genomic changes in *F*. *×bohemica* assuming that this taxon retains the ability to reproduce sexually and if so, what are the precise factors that may limit such sexual reproduction and move the population towards clonal spread.

## Supporting Information

S1 Table AFLP primer combinations used in the experiment. X–primer combinations used in the analysis of heterogeneous and homogeneous populations.
(PDF)

S2 Table Number of polymorphic and monomorphic AFLP loci compiled in a binary data matrix. (XLS)

S3 Table The level of polymorphism between *Fallopia* populations from the introduced and native ranges. Codes of populations–see Table 1. FJ–*Fallopia japonica*; FB–*F*. ×*bohemica*; FS–*F*. *sachalinensis*. ^a^ The levels of polymorphism were calculated after pairwise analyses of individuals from each taxon.
(PDF)

S4 Table The values of maximum likelihood-based hybrid index (h) for each of *F*. *× bohemica* individual from the population in Jasieniczanka River (‘heterogeneous’ stand composed of three taxa). Expected h = 0 for *F*. *sachalinensis* parental species; expected h = 1 for *F*. *japonica* parental species; lnL–likelihood values.
(PDF)

## References

[1] Child LE, Wade MP. The Japanese Knotweed Manual The Management and Control of an Invasive Alien Weed. 2000 Packard Publishing Limited, Chichester.

[2] Bailey JP. Japanese Knotweeds s.l. at home and abroad In: Child L, Brock JH, Brundu G, Prach K, Pyšek P, Wade PM & Williamson M, editors. Plant Invasions: Ecological Threats and Management Solutions. Backhuys, Leiden; 2003 pp. 183–196.

[3] Alberternst B, Böhmer HJ. NOBANIS–Invasive Alien Species Fact Sheet–Fallopia japonica.–From: Online Database of the European Network on Invasive Alien Species–NOBANIS. 2011. Available: http://www.nobanis.org. Accessed 23 Jul 2016.

[4] Jäger EJ. Die Gesamtareale von *Reynoutria japonica* Houtt. und *R*. *sachalinensis* (F. Schmidt) Nakai, ihre klimatische Interpretation und Daten zur Ausbreitungsgeschichte. Schr.–R.f. Vegetationskde. Sukopp–Festschrift. 1995; 27: 395–403.

[5] Bailey JP, Conolly AP. Prize-winners to pariahs–a history of Japanese knotweed s.l. (Polygonaceae) in the British Isles. Watsonia. 2000; 23: 93–110.

[6] Tokarska-Guzik B. The establishment and spread of alien plant species (kenophytes) in the flora of Poland University of Silesia, Katowice; 2005. No 2372.

[7] Bailey JP. The Japanese knotweed invasion viewed as a vast unintentional hybridization experiment. Heredity. 2013; 105: 105–110. 10.1038/hdy.2012.98 PMC3554452 23211787

[8] Chrtek J, Chrtková A. *Reynoutria* × *bohemica*, a new hybrid from the dock family. Časopis Nàrodniho Muzea v Praze. Ser. Nat. 1983; 152: 120.

[9] Beerling DJ, Bailey JP, Conolly AP. *Fallopia japonica* (Houtt.) Ronse Decraene (*Reynoutria japonica* Houtt.; *Polygonum cuspidatum* Sieb. Zucc.). J Ecol. 1994; 82: 959–979.

[10] Barney JN, Tharayil N, Di Tommaso A, Bhowmik PC. The biology of invasive alien plants in Canada. 5. *Polygonum cuspidatum* Sieb. & Zucc. [= *Fallopia japonica* (Houtt.) Ronse Decr.]. Can J Plant Sci. 2006; 86: 887–905.

[11] Bailey JP, Bimová K, Mandák B. The potential role of polyploidy and hybridization in the further evolution of the highly invasive *Fallopia* taxa in Europe. Ecol Res. 2007; 22: 920–928.

[12] Bailey JP, Bímová K, Mandák B. Asexual spread versus sexual reproduction and evolution in Japanese Knotweed s.l. sets the stage for the ‘Battle of the Clones’. Biol Invasions. 2009; 11: 1189–1203.

[13] Saldaña A, Fuentes N, Pfanzelt S. *Fallopia japonica* (Houtt.) Ronse Decr. (Polygonaceae): a new record for the alien flora of Chile. Gayana Bot. 2009; 66: 283–285.

[14] Sukopp H, Starfinger U. *Reynoutria sachalinensis* in Europe and in the Far East: a comparison of the species ecology in its native and adventive distribution range In: Pyšek P, Prach K, Rejmánek M & Wade M, editors. Plant invasions: general aspects and special problems. SPB Academic Publishing, Amsterdam, The Netherlands; 1995 pp. 151–159.

[15] Owen SJ. Ecological weeds on conservation land in New Zealand: a database; 1996 Department of Conservation, Wellington, New Zealand: DOC Science Publications Accessed: http://www.hear.org/weedlists/other_areas/nz/nzecoweeds.htm.

[16] Gibbs Russell GE, Welman WG, Reitief E, Immelman KL, Germishuizen G, Pienaar BJ, et al List of species of southern African plants. Mem Bot Surv South Africa. 1987; 2(1,2): 1–152, 1–270.

[17] Pyšek P, Prach K. Plant invasions and the role of riparian habitats: a comparison of four species alien to central Europe. J Biogeogr. 1993; 20: 413–420.

[18] Richards CL, Walls RL, Bailey JP, Parameswaran R, George T, Pigliucci M. Plasticity in salt tolerance traits allows for invasion of novel habitat by Japanese knotweed s.l. (*Fallopia japonica* and *F*. × *bohemica*, Polygonaceae). Am J Bot. 2008; 95: 931–942. 10.3732/ajb.2007364 21632416

[19] Urgenson LS, Reichard SH, Halpern CB. Community and ecosystem consequences of giant knotweed (*Polygonum sachalinense*) invasion into riparian forests of western Wahington, USA. Biol Conserv. 2009; 142: 1536–1541.

[20] Tokarska-Guzik B, Dajdok Z, Zając M, Zając A, Urbisz Al, Danielewicz W, et al Rośliny obcego pochodzenia w Polsce ze szczególnym uwzględnieniem gatunków inwazyjnych (Alien plants in Poland with particular reference to invasive species). Generalna Dyrekcja Ochrony Środowiska, Warszawa, 2012; pp. 1–197 [in Polish].

[21] Pyšek P, Genovesi P, Pergl J, Monaco A, Wild J. Plant invasions of protected areas in Europe: an old continent facing new problems In: Foxcroft LC, Pyšek P, Richardson DM & Genovesi P, editors. Plant invasions in protected areas: patterns, problems and challenges. Springer, Dordrecht; 2013 pp. 209–240.

[22] Mandák B, Pyšek P, Lysák M, Suda J, Krahulcová A, Bimová K.Variation in DNA-ploidy levels of *Reynoutria* taxa in the Czech Republic. Ann Bot. 2003; 92: 265–272. 12876190 10.1093/aob/mcg141 PMC4243663

[23] Ellstrand NC, Schierenbeck KA. Hybridization as a stimulus for the evolution of invasiveness in plants? PNAS. 2000; 97: 7043–7050. 10860969 10.1073/pnas.97.13.7043 PMC34382

[24] Tiébré MS, Vanderhoeven S, Saad L, Mahy G. Hybridization and sexual reproduction in the invasive alien *Fallopia* (Polygonaceae) complex in Belgium. Ann Bot. 2007; 99(1): 193–203. 17210609 10.1093/aob/mcl242 PMC2802983

[25] Vilà M., Weber E., Carla M. D’Antonio. Conservation implications of invasion by plant hybridization. Biol Invasions. 2000; 2: 207–217.

[26] Parepa M, Fischer M, Krebs C, Bossdorf O. Hybridization increases invasive knotweed success. Evol Appl. 2014; 7: 413–420. 10.1111/eva.12139 24665343 PMC3962301

[27] Genton BJ, Shykoff JA, Giraud T. High genetic diversity in French invasive populations of common ragweed, *Ambrosia artemisiifolia*, as a result of multiple sources of introduction. Mol Ecol. 2005; 14: 4275–4285. 16313592 10.1111/j.1365-294X.2005.02750.x

[28] Rosenthal DM, Ramakrishnan A, Cruzan MB. Evidence for multiple sources of invasion and intraspecific hybridization in *Brachypodium sylvaticum* (Hudson) Beauv. in North America. Mol Ecol. 2008; 17: 4657–4669. 10.1111/j.1365-294X.2008.03844.x 18627455

[29] Loveless MD, Hamrick JL. Ecological determinants of genetic structure in plant populations. Annu Rev Ecol Evol Syst. 1984; 15: 65–95.

[30] Nybom H. Comparison of different nuclear DNA markers for estimating intraspecific genetic diversity in plants. Mol Ecol. 2004; 13: 1143–1155. 15078452 10.1111/j.1365-294X.2004.02141.x

[31] Novak SJ, Mack RN. Genetic bottlenecks in alien plant species: influences of mating systems and introduction dynamics In: Sax DF, Stachowicz JJ, Gaines SD, editors. Species Invasions, insights into ecology, evolution and biogeography. Sunderland MA: Sinauer & Associates; 2005 pp. 201–228.

[32] Dlugosch KM, Hays C. Genotypes on the move some things old and some things new shape the genetics of colonization during species invasions. Mol Ecol. 2008; 17: 4583–4585. 10.1111/j.1365-294X.2008.03932.x 18992002

[33] Lembicz M, Piszczałka P, Grzybowski T, Woźniak M, Jarmołowski A, Borkowska L, et al Microsatellite identification of ramet genotypes in a clonal plant with phalanx growth: The case of *Cirsium rivulare* (Asteraceae). Flora. 2011; 206: 792–798.

[34] Amsellem L, Noyer JL, Le Bourgeois T, Hossaert-Mckey M. Comparison of genetic diversity of the invasive weed *Rubus alceifolius* Poir. (Rosaceae) in its native range and in areas of introduction, using amplified fragment length polymorphism (AFLP) markers. Mol Ecol. 2000; 9: 443–455. 10736047 10.1046/j.1365-294x.2000.00876.x

[35] Hery P, Le Lay G, Goudet J, Guisan A, Jahodová S, Besnard G.Reduced genetic diversity, increased isolation and multiple introductions of invasive giant hogweed in the western Swiss Alps. Mol Ecol. 2009;18: 2819–2831. 10.1111/j.1365-294X.2009.04237.x 19500248

[36] Zhang YY, Zhang DY, Barrett SCH. Genetic uniformity characterizes the invasive spread of water hyacinth (*Eichhornia crassipes*), a clonal aquatic plant. Mol Ecol. 2010; 19: 1774–1786. 10.1111/j.1365-294X.2010.04609.x 20529068

[37] Matesanz S, Theiss KE, Holsinger KE, Sultan SE. Genetic diversity and population structure in *Polygonum cespitosum*: Insights to an ongoing plant invasion. PLoS ONE. 2014; 9(4): e93217 10.1371/journal.pone.0093217 24695495 PMC3973574

[38] Stout JC, Duffy KJ, Egan PA, Harbourne M, Hodkinson TR. Genetic diversity and floral width variation in introduced and native populations of a long-lived woody perennial. AoB Plants. 2015; 7, plu087. 10.1093/aobpla/plu087 PMC4323518 25527475

[39] Sakai A, Allendorf FW, Holt JS, Lodge DM, Molofsky J, With KA, et al The population biology of invasive species. Annu Rev Ecol Evol Syst. 2001; 32 305–332.

[40] Taylor DR, Keller SR. Historical range expansion determines the phylogenetic diversity introduced during contemporary species invasion. Evolution. 2007; 61: 334–345. 17348944 10.1111/j.1558-5646.2007.00037.x

[41] Dlugosch KM, Parker IM. Invading populations of an ornamental shrub show rapid life history evolution despite genetic bottlenecks. Ecol Lett. 2008; 11: 701–709. 10.1111/j.1461-0248.2008.01181.x 18410377

[42] Hollingsworth ML, Hollingsworth PM, Jenkins GI, Bailey JP, Ferris C. The use of molecular markers to study patterns of genotypic diversity in some invasive alien *Fallopia* spp. (Polygonaceae). Mol Ecol. 1998; 7: 1681–1691.

[43] Tiébré MS, Bizoux JP, Hardy OJ, Bailey JP, Mahy G. Hybridization and morphogenetic variation in the invasive alien *Fallopia* (Polygonaceae) complex in Belgium. Am J Bot. 2007; 94: 1900–1910. 10.3732/ajb.94.11.1900 21636383

[44] Krebs C, Mahy G, Matthies D, Schaffner U, Tiébré MS, Bizoux J. Taxa distribution and RAPD markers indicate different origin and regional differentiation of hybrids in the invasive *Fallopia* complex in central-western Europe. Plant Biology. 2010; 12: 215–223. 10.1111/j.1438-8677.2009.00219.x 20653904

[45] Gammon MA, Grimsby JL, Tsirelson D, Kesseli R. Molecular and morphological evidence reveals introgression in swarms of the invasive taxa *Fallopia japonica*, *F*. *sachalinensis* and *F*. *× bohemica* (Polygonaceae) in the United States. Am J Bot. 2007; 94: 948–956. 10.3732/ajb.94.6.948 21636463

[46] Grimsby JL, Tsirelson D, Gammon MA, Kesseli R. Genetic diversity and clonal vs sexual reproduction in *Fallopia* spp. (Polygonaceae). Am J Bot. 2007; 94: 957–996. 10.3732/ajb.94.6.957 21636464

[47] Bzdęga K, Janiak A, Tarłowska S, Kurowska M, Tokarska-Guzik B, Szarejko I. Unexpected genetic diversity of *Fallopia japonica* from Central Europe revealed after AFLP analysis. Flora. 2012; 207: 636–645.

[48] Gaskin JF, Schwarzländer M, Grevstad FS, Haverhals MA, Bourchier RS, Miller T. Extreme differences in population structure and genetic diversity for three invasive congeners: knotweeds in western North America. Biol Invasions. 2014; 16: 2127–2136.

[49] Mandák B, Bímová K, Pyšek P, Štepánek J, Placková I. Isoenzyme diversity in *Reynoutria* taxa: escape from sterility by hybridization. Plant Syst Evol. 2005; 253: 219–230.

[50] Bailey JP, Stace CA. Chromosome number, morphology, pairing and DNA values of species and hybrids in the genus *Fallopia* (Polygonaceae). Plant Syst Evol. 1992; 180: 29–52.

[51] Suda J, Trávníček P, Mandák B, Berchová-Bimová K. Genome size as a marker for identifying the invasive alien taxa in *Fallopia* section *Reynoutria*. Preslia. 2010; 82: 97–106.

[52] Komuro S, Endo R, Shikata K, Kato A. Genomic and chromosomal distribution patterns of various repeated DNA sequences in wheat revealed by a fluorescence *in situ* hybridization procedure. Genome. 2013; 56: 131–137. 10.1139/gen-2013-0003 23659696

[53] Richards, Moorehead & Laing Ltd. Japanese Knotweed (Reynoutria japonica) in Wales. Report to the Welsh Development Agency, Cardiff; 1990.

[54] Doyle JJ, Doyle JK. A rapid DNA isolation procedure for small quantities of fresh leaf tissue. Phytochem Bull. 1987; 19: 11–15.

[55] Vos P, Hogers R, Bleeker M, Reijans M, van de Lee T, Hornes M, et al AFLP: a new technique for DNA fingerprinting. Nucleic Acids Research. 1995; 23: 4407–4414. 7501463 10.1093/nar/23.21.4407 PMC307397

[56] Pielou EC. An introduction to mathematical ecology Wiley Interscience. John Wiley and Sons, New York; 1969.

[57] Vekemans X, Beauwens T, Lemaire M, Roldan-Ruiz I. Data from amplified fragment length polymorphism (AFLP) markers show indication of size homoplasy and of a relationship between degree of homoplasy and fragment size. Mol Ecol. 2002; 11: 139–151. 11903911 10.1046/j.0962-1083.2001.01415.x

[58] Excoffier L, Smouse PE, Quattro JM. Analysis of molecular variance inferred from metric distances among DNA haplotypes: application to human mitochondrial DNA restriction data. Genetics. 1992; 131: 479–491. 1644282 10.1093/genetics/131.2.479 PMC1205020

[59] Felsenstein J. PHYLIP, Phylogeny Inference Package, version 3.6 Distributed by the author. Department of Genomic Sciences, University of Washington, Seattle; 2004.

[60] Choi JH, Jung HY, Kim HS, Cho HG. PhyloDraw: A phylogenetic tree drawing system. Bioinformatics. 2000; 16: 1056–1058. 11159323 10.1093/bioinformatics/16.11.1056

[61] Schlüter PM, Harris SA. Analysis of multilocus fingerprinting data sets containing missing data. Mol Ecol Notes. 2006; 6: 569–572.

[62] Błocka-Wandas M, Sliwinska E, Grabowska-Joachimiak A, Musial K, Joachimiak AJ. Male gametophyte development and two different DNA classes of pollen grains in *Rumex acetosa* L., a plant with an XX/XY_1_Y_2_ sex chromosome system and a female-biased sex ratio. Sex Plant Reprod. 2007; 20: 171–180.

[63] Gerlach WL, Dyer TA. Sequence organisation of the repeating units in the nucleus of wheat which contain 5S rRNA genes. Nucleic Acids Res. 1980; 8: 4851–4865. 7443527 10.1093/nar/8.21.4851 PMC324264

[64] Unfried I, Gruendler P. Nucleotide sequence of the 5.8S and 25S rRNA genes and the internal transcribed spacers from *Arabidopsis thaliana*. Nucleic Acids Res. 1990; 18: 4011 2100998 10.1093/nar/18.13.4011 PMC331127

[65] Grabowska-Joachimiak A, Kula A, Książczyk T, Chojnicka J, Sliwinska E, Joachimiak A. Chromosome landmarks and autosome-sex chromosome translocations in *Rumex hastatulus*, a plant with XX/XY1Y2 sex chromosome system. Chromosome Res. 2015; 23: 187–197. 10.1007/s10577-014-9446-4 25394583 PMC4430600

[66] Książczyk T, Zwierzykowska E, Molik K, Taciak M, Krajewski P, Zwierzykowski Z. Genome-dependent chromosome dynamics in three successive generations of the allotetraploid *Festuca pratensis* × *Lolium perenne* hybrid. Protoplasma. 2015; 252: 985–996. 10.1007/s00709-014-0734-9 25480732 PMC4491343

[67] Richards CL, Schrey AW, Pigliucci M, Vellend M. Invasion of diverse habitats by few Japanese knotweed genotypes is correlated with epigenetic differentiation. Ecol Lett. 2012; 15: 1016–1025. 10.1111/j.1461-0248.2012.01824.x 22731923

[68] Fukui S, Araki KS. Spatial niche facilitates clonal reproduction in seed plants under temporal disturbance. PLoS ONE. 2014; 9(12): e116111 10.1371/journal.pone.0116111 25549330 PMC4280169

[69] Buhk C, Thielsch A. Hybridisation boosts the invasion of an alien species complex: Insights into future invasiveness. PPEES. 2015; 17: 274–283.

[70] Du ZY, Yang CF, Cheng JM, Guo YH. Nuclear and chloroplast DNA sequences data support the origin of *Potamogeton intortusifolius* J.B. He in China as a hybrid between *P*. *perfoliatus* Linn. and *P*. *wrightii* Morong. Aquat Bot. 2009; 91: 47–50.

[71] Wan T, Zhang XL, Gregan J, Zhang Y, Guo P, Guo Y. A dynamic evolution of chromosome in subgenus *Potamogeton* revealed by physical mapping of rDNA loci detection. Plant Syst Evol. 2012; 298: 1195–1210.

[72] Garcia S, Lim KY, Chester M, Garnatje T, Pellicer J, Valle`s J, et al Linkage of 35S and 5S rRNA genes in *Artemisia* (family Asteraceae): first evidence from angiosperms. Chromosoma. 2009; 118: 85–97. 10.1007/s00412-008-0179-z 18779974

[73] Soltis DE, Soltis PS. Polyploidy: recurrent formation and genome evolution. Trends Ecol Evol. 1999; 14: 348–352. 10441308 10.1016/s0169-5347(99)01638-9

[74] Hasterok R, Wolny E, Hosiawa M, Kowalczyk M, Kulak-Książczyk S, Książczyk T, et al Comparative analysis of rDNA distribution in chromosomes of various species of Brassicaceae. Ann Bot. 2006; 97: 205–216. 16357054 10.1093/aob/mcj031 PMC2803362

[75] Pedrosa-Harand A, de Almeida CCS, Mosiolek M, Blair MW, Schweizer D, Guerro M. Extensive ribosomal DNA amplification during Andean common bean (*Phaseolus vulgaris* L.) evolution. Theor Appl Genet. 2006; 112: 924–933. 16397788 10.1007/s00122-005-0196-8

[76] Mishima M, Ohmido N, Fukui K, Yahara T. Trends in site-number change of rDNA loci during polyploid evolution in *Sanguisorba* (Rosaceae). Chromosoma. 2002; 110: 550–558. 12068972 10.1007/s00412-001-0175-z

[77] Wendel JF, Schnabel A, Seelanan T. Bidirectional interlocus concerted evolution following allopolyploid speciation in cotton (*Gossypium*). PNAS. 1995; 92: 280–284. 7816833 10.1073/pnas.92.1.280 PMC42862

[78] Shaked H, Kashkush K, Ozkan H, Feldman M, Levy AA. Sequence elimination and cytosine methylation are rapid and reproducible responses of the genome to wide hybridization and allopolyploidy in wheat. The Plant Cell. 2001; 13: 1749–1759. 11487690 10.1105/TPC.010083 PMC139131

[79] Książczyk T, Kovarík A, Eber F, Huteau V, Khaitova L, Tesarikova Z, et al Immediate unidirectional epigenetic reprogramming of NORs occurs independently of rDNA rearrangements in synthetic and natural form of a polyploid species *Brassica napus*. Chromosoma. 2011; 120: 557–571. 10.1007/s00412-011-0331-z 21785942

[80] Xiong Z, Gaeta R, Pires C. Homoeologous shuffling and chromosome compensation maintain genome balance in resynthesized allopolyploid *Brassica napus*. PNAS. 2011; 108: 7908–7913. 10.1073/pnas.1014138108 21512129 PMC3093481

[81] Datson PM, Murray BG. Ribosomal DNA locus evolution in *Nemesia*: transposition rather than structural rearrangement as the key mechanism? Chromosome Res. 2006; 14: 845–857. 17195054 10.1007/s10577-006-1092-z

[82] Malinska H, Tate JA, Matyasek R, Leitch AR, Soltis DE, Soltis PS, et al Similar patterns of rDNA evolution in synthetic and recently formed natural populations of *Tragopogon* (Asteraceae) allotetraploids. BMC Evol Biol. 2010; 10: 291 10.1186/1471-2148-10-291 20858289 PMC2955031

