# Supplementary material for: A Survey of Genetic Variation and Genome Evolution within the Invasive Fallopia Complex
Source: PLoS One. 2016 Aug 30;11(8):e0161854. doi: 10.1371/journal.pone.0161854 (PMC5004975; doi:10.1371/journal.pone.0161854)
Supplement: S1 Table — X–primer combinations used in the analysis of heterogeneous and homogeneous populations. (PDF) [file pone.0161854.s001.pdf]

S1 Table. AFLP primer combinations used in the experiment.

Primers	M-CAT	M-CCA	M-CTA	M-CTC
**E-ACA**	X		X	X
**E-ACC**	X		X	
**E-ACT**	X			X
**E-AGC**	X			X
**E-AGG**		X		

X – primer combinations used in the analysis of heterogeneous and homogeneous populations.
