# Supplementary material for: A Survey of Genetic Variation and Genome Evolution within the Invasive Fallopia Complex
Source: PLoS One. 2016 Aug 30;11(8):e0161854. doi: 10.1371/journal.pone.0161854 (PMC5004975; doi:10.1371/journal.pone.0161854)
Supplement: S3 Table — Codes of populations–see Table 1. FJ–Fallopia japonica; FB–F. ×bohemica; FS–F. sachalinensis. a The levels of polymorphism were calculated after pairwise analyses of individuals from each taxon. (PDF) [file pone.0161854.s003.pdf]

S3 Table. The level of polymorphism between *Fallopia* populations from the introduced and native ranges.

Compared populations		DNA polymorphism (%)[a]	
		Range	Mean value
		**introduced range**	
PL_CDJ_FJ	PL_CDJ_FB	5.89 – 9.63	7.89
	PL_CDJ_FS	9.01 – 10.64	10.22
	PL_CDB_FJ	4.32 – 5.48	4.77
	PL_CDB_FJ_h	4.79 – 6.66	4.91
	PL_CDB_FB	5.04 – 8.75	6.48
PL_CDJ_FS	PL_CDB_FJ	9.96 – 10.55	10.29
	PL_CDB_FB	10.85 – 13.84	11.00
	PL_CDB_FJ_h	10.86 – 11.16	10.92
PL_CDB_FJ	PL_CDB_FJ_h	2.74 – 3.03	2.76
	PL_CDB_FB	4.50 – 5.77	4.82
PL_CDB_FB	PL_CDB_FJ_h	5.41 – 5.79	5.43
		**native range**	
JP_It_FJ	JP_Os_FJ	7.81 – 9.73	8.74
JP_It_FJ	JP_To_FS	23.95 – 25.83	24.85
JP_It_FJ	JP_Ob_FS	24.10 – 26.25	25.22
JP_Os_FJ	JP_To_FS	21.87 – 22.78	22.34
JP_Os_FJ	JP_Ob_FS	22.17 – 23.00	22.72
JP_To_FS	JP_Ob_FS	3.02 – 4.41	3.75
		**introduced vs. native range**	
PL_CDJ_FJ	JP_It_FJ	10.67 – 13.82	12.50
	JP_Os_FJ	9.04 – 10.67	10.21
	JP_To_FS	18.34 – 20.27	19.79
	JP_Ob_FS	18.64 – 20.62	20.28
PL_CDJ_FS	JP_It_FJ	16.39 – 19.02	17.67
	JP_Os_FJ	15.13 – 15.75	15.38
	JP_To_FS	19.08 – 20.40	19.73
	JP_Ob_FS	19.39 – 20.88	20.27
PL_CDB_FJ	JP_It_FJ	11.36 – 14.14	12.24
	JP_Os_FJ	9.72 – 10.30	9.92
	JP_To_FS	19.61 – 20.51	20.13
	JP_Ob_FS	9.72 – 21.13	20.47
PL_CDB_FJ_h	JP_It_FJ	11.98 – 14.22	12.78
	JP_Os_FJ	10.61 – 10.93	10.80
	JP_To_FS	19.88 – 20.33	20.08
	JP_Ob_FS	20.03 – 21.23	20.84

PL_CDB_FB+	JP_It_FJ	12.24 – 16.37	13.15
	JP_Os_FJ	11.16 – 12.22	11.37
	JP_To_FS	19.43 – 22.22	19.81
	JP_Ob_FS	19.73 – 23.39	20.31

Codes of populations – see Table 1. FJ – *Fallopia japonica*; FB – *F.* ×*bohemica*; FS – *F. sachalinensis.*
[a] The levels of polymorphism were calculated after pairwise analyses of individuals from each taxon.
