# Supplementary material for: A Survey of Genetic Variation and Genome Evolution within the Invasive Fallopia Complex
Source: PLoS One. 2016 Aug 30;11(8):e0161854. doi: 10.1371/journal.pone.0161854 (PMC5004975; doi:10.1371/journal.pone.0161854)
Supplement: S4 Table — Expected h = 0 for F. sachalinensis parental species; expected h = 1 for F. japonica parental species; lnL–likelihood values. (PDF) [file pone.0161854.s004.pdf]

S4 Table. The values of maximum likelihood-based hybrid index (h) for each of *F.* × *bohemica* individual from the population in Jasieniczanka River (‘heterogeneous’ stand composed of three taxa).

Individual	h	Lower Bound	Upper Bound	lnL
PL_CDJ_FB_1	0.583	0.476	0.688	-42.001
PL_CDJ_FB_2	0.583	0.476	0.688	-42.001
PL_CDJ_FB_3	0.587	0.482	0.688	-52.392
PL_CDJ_FB_4	0.577	0.472	0.680	-47.920
PL_CDJ_FB_5	0.593	0.487	0.697	-46.348
PL_CDJ_FB_6	0.587	0.477	0.694	-34.624
PL_CDJ_FB_7	0.640	0.533	0.741	-48.713
PL_CDJ_FB_8	0.622	0.516	0.724	-50.561
PL_CDJ_FB_9	0.614	0.507	0.717	-46.047
PL_CDJ_FB_10	0.599	0.492	0.703	-45.507
PL_CDJ_FB_11	0.596	0.487	0.701	-39.813
PL_CDJ_FB_12	0.595	0.490	0.697	-50.074
PL_CDJ_FB_13	0.658	0.552	0.758	-42.299
PL_CDJ_FB_14	0.613	0.509	0.712	-58.870
PL_CDJ_FB_15	0.613	0.509	0.712	-58.870
PL_CDJ_FB_16	0.597	0.495	0.696	-60.557
PL_CDJ_FB_17	0.522	0.416	0.628	-42.978
PL_CDJ_FB_18	0.538	0.431	0.644	-41.720
PL_CDJ_FB_19	0.538	0.431	0.644	-41.720
PL_CDJ_FB_20	0.538	0.431	0.644	-41.720
PL_CDJ_FB_21	0.603	0.496	0.705	-46.565
PL_CDJ_FB_22	0.528	0.423	0.633	-43.376
PL_CDJ_FB_23	0.526	0.419	0.634	-37.066
PL_CDJ_FB_24	0.526	0.419	0.634	-37.066
PL_CDJ_FB_25	0.526	0.419	0.634	-37.066
PL_CDJ_FB_26	0.526	0.419	0.634	-37.066
PL_CDJ_FB_27	0.526	0.419	0.634	-37.066
PL_CDJ_FB_28	0.526	0.419	0.634	-37.066
PL_CDJ_FB_29	0.526	0.419	0.634	-37.066
PL_CDJ_FB_30	0.526	0.419	0.634	-37.066

Expected h=0 for *F. sachalinensis* parental species; expected h=1 for *F. japonica* parental species; lnL – likelihood values.
